# Effect of nocturnal oxygen therapy on exercise performance of COPD patients at 2048 m: data from a randomized clinical trial

**DOI:** 10.1038/s41598-021-98395-w

**Published:** 2021-10-13

**Authors:** Sophia Gutweniger, Tsogyal D. Latshang, Sayaka S. Aeschbacher, Fabienne Huber, Deborah Flueck, Mona Lichtblau, Stefanie Ulrich, Elisabeth D. Hasler, Philipp M. Scheiwiller, Silvia Ulrich, Konrad E. Bloch, Michael Furian

**Affiliations:** Department of Respiratory Medicine, University Hospital of Zurich, Raemistrasse 100, 8091 Zurich, Switzerland

**Keywords:** Randomized controlled trials, Outcomes research, Chronic obstructive pulmonary disease

## Abstract

This trial evaluates whether nocturnal oxygen therapy (NOT) during a stay at 2048 m improves altitude-induced exercise intolerance in lowlanders with chronic obstructive pulmonary disease (COPD). 32 lowlanders with moderate to severe COPD, mean ± SD forced expiratory volume in the first second of expiration (FEV_1_) 54 ± 13% predicted, stayed for 2 days at 2048 m twice, once with NOT, once with placebo according to a randomized, crossover trial with a 2-week washout period at < 800 m in-between. Semi-supine, constant-load cycle exercise to exhaustion at 60% of maximal work-rate was performed at 490 m and after the first night at 2048 m. Endurance time was the primary outcome. Additional outcomes were cerebral tissue oxygenation (CTO), arterial blood gases and breath-by-breath measurements (http://www.ClinicalTrials.gov NCT02150590). Mean ± SE endurance time at 490 m was 602 ± 65 s, at 2048 m after placebo 345 ± 62 s and at 2048 m after NOT 293 ± 60 s, respectively (P < 0.001 vs. 490 m). Mean difference (95%CI) NOT versus placebo was − 52 s (− 174 to 70), P = 0.401. End-exercise pulse oximetry (SpO_2_), CTO and minute ventilation (${\dot{\text{V}}}_{{\text{E}}}$) at 490 m were: SpO_2_ 92 ± 1%, CTO 65 ± 1%, ${\dot{\text{V}}}_{{\text{E}}}$ 37.7 ± 2.0 L/min; at 2048 m with placebo: SpO_2_ 85 ± 1%, CTO 61 ± 1%, ${\dot{\text{V}}}_{{\text{E}}}$ 40.6 ± 2.0 L/min and with NOT: SpO_2_ 84 ± 1%; CTO 61 ± 1%; ${\dot{\text{V}}}_{{\text{E}}}$ 40.6 ± 2.0 L/min (P < 0.05, SpO_2_, CTO at 2048 m with placebo vs. 490 m; P = NS, NOT vs. placebo). Altitude-related hypoxemia and cerebral hypoxia impaired exercise endurance in patients with moderate to severe COPD and were not prevented by NOT.

## Introduction

Chronic obstructive pulmonary disease (COPD) is characterized by persistent airflow limitation due to chronic inflammation of the airways (obstructive bronchitis and bronchiolitis) and destruction of the lung parenchyma (emphysema)^1^. Patients with COPD suffer already near sea level from reduced physical performance due to disease-related excessive dyspnea sensation, gas exchange impairments leading to pronounced hypoxemia and ventilatory constraints^2^. Although COPD is incurable, pharmacological therapy and supplemental oxygen improve symptoms and many patients can live for decades with COPD^1^. With an estimated prevalence of 8–15% of the global population, patients with COPD are assumed to be among the estimated 300–350 million annual winter season visitors, who travel by car or gondola to locations up to 3000 m without relevant physical effort^1,3^.

The lower barometric pressure at altitude causes a decrease in the inspired and alveolar oxygen partial pressure and reduces systemic oxygen availability. A recent trial in 40 patients with moderate to severe COPD travelling to 2590 m found a 54% reduction in cycling endurance time as well as pronounced nocturnal hypoxemia, sleep-disordered breathing and acute mountain sickness compared to near sea level^4–6^. Exercise-limiting factors were arterial hypoxemia, cerebral hypoxia, excessive dyspnea sensation and worse ventilatory equivalents for O_2_ uptake and CO_2_ output compared to 490 m^4^.

Oxygen supplementation during exercise has been shown to improve endurance time near sea level in healthy individuals^7^, patients with pulmonary hypertension^8^ and in patients with COPD^9^. However, as the administration of daytime oxygen is impractical and cumbersome when performing outdoor activities in the mountains, nocturnal oxygen therapy (NOT) might be a better alternative. NOT might mitigate nocturnal hypoxemia, sleep-related disturbances and might disrupt the progression of acute mountain sickness (highest prevalence after the first night at high altitude), therefore, NOT might have an indirect beneficial effect on next-day exercise performance. Indeed, the analysis of the primary outcome of this study showed that NOT improves nocturnal oxygenation, breathing stability and subjective sleep quality compared to placebo in patients with COPD staying overnight at 2048 m^10^. Whether these favorable effects persist over the day remains to be elucidated.

Therefore, the purpose of this analysis of secondary outcomes was to test the hypothesis that the previously shown improvements of NOT augment next-day exercise performance in patients with COPD spending a night at 2048 m compared to placebo intervention. Furthermore, the current trial investigated exercise-limiting factors at high versus low altitude, and the physiological effects of NOT compared to placebo.

## Methods

### Study design and participants

The current study was nested within a randomized, placebo-controlled, cross-over trial conducted from January 1 to October 31, 2014, evaluating the effects of NOT on sleep, nocturnal oxygen saturation and altitude-related adverse health effects (ARAHEs) (ClinicalTrials.gov NCT02143609). Baseline characteristics of participants and results from ARAHEs have been published recently^10^. Data of the current study focused on exercise performance and have not been reported elsewhere. Patients underwent baseline examinations at 490 m (University Hospital of Zurich, Switzerland) and performed the same evaluations over the course of 2 sojourns at 2048 m (St. Moritz, Switzerland) with a 2-week washout period < 800 m in-between altitude stays and in randomized order. In the nights at 2048 m, NOT or placebo (ambient air) were administered at a flow rate of 3 L/min per nasal cannula. The sequence of altitude exposure and treatment was randomized. Before measurements, patients were requested to refrain from smoking, however, smoking was not prohibited and controlled. The Cantonal Ethics Committee Zurich (EK-2013-0088) approved the protocol and patients gave their written informed content. The trial followed the CONSORT reporting guidelines and all experiments were performed in accordance with relevant guidelines and regulations.

Eligible participants were patients with moderate to severe COPD, Global Initiative for Obstructive Lung Disease grade 2–3 (forced expiratory volume in the first second of expiration [FEV_1_]/forced vital capacity [FVC] < 0.7 and FEV_1_ 30–80% predicted), aged 18 to 75 years, male or female and living < 800 m. Exclusion criteria were COPD exacerbation within 4 months before the study, hypoxemia of a peripheral oxygen saturation (SpO_2_) < 92% at 490 m, any uncontrolled cardiovascular disease, previous altitude intolerance (< 2600 m) or exposure to altitude > 1500 m for > 2 days within the last 4 weeks before the study.

### Randomization and interventions

**Figure 1:**
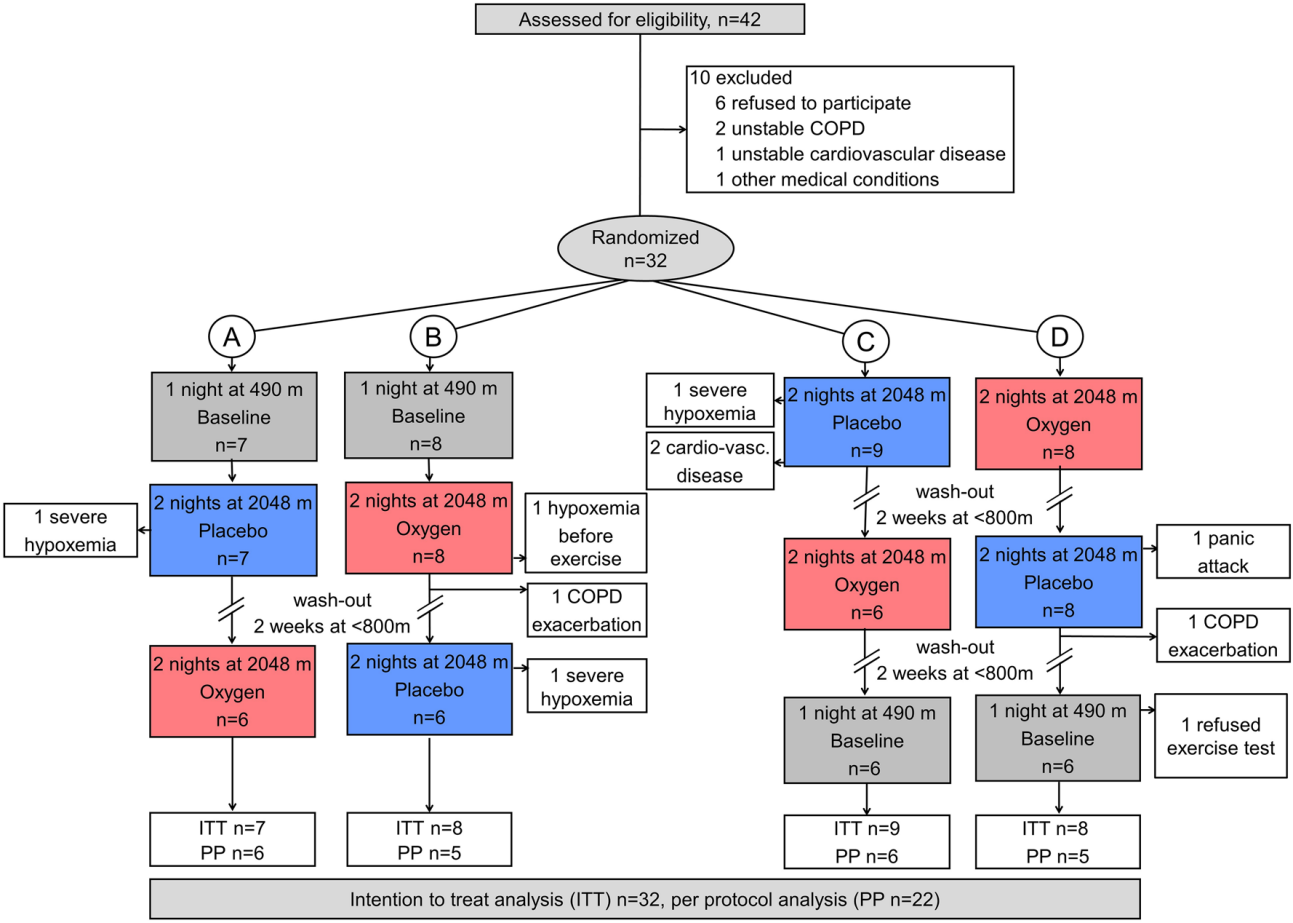
CONSORT Flowchart. Altitude allocation sequence A–D and order of intervention (oxygen or placebo) at 2048 m were randomized. After evaluations at 2048 m, a 2-week washout period at < 800 m was applied to avoid carry-over effects. *COPD* chronic obstructive pulmonary disease, *PP* per-protocol, *ITT* intention-to-treat.

Patients were randomized in balanced blocks of 4 to sequences of altitude exposure and treatment by letting them draw a sealed envelope with one of the following allocations (Fig. 1): (A) 490 m—2048 m placebo—2048 m NOT; (B) 490 m—2048 m NOT—2048 m placebo; (C) 2048 m placebo—2048 m NOT—490 m; (D) 2048 m NOT—2048 m placebo—490 m. Patients traveled by train and car within 3 h from 490 to 2048 m. Between altitude sojourns, a washout period < 800 m of at least 2-weeks was interposed. During two nights at 2048 m, patients were wearing nasal prongs connected to a concentrator (EverFlow, Philips Respironics, Switzerland) delivering oxygen (fraction of inspired oxygen [FiO_2_] 1.0) or placebo (ambient air) at a flow rate of 3 L/min. Patients were blinded for the intervention by placing concentrators in a separate room. Investigators performing the data analysis were also blinded to intervention and exposure sequence. For safety reasons, patients’ nocturnal oxygen saturation was monitored by investigators.

### Assessments

At 490 m and after the first night of each stay at 2048 m, patients underwent a cycle constant work-rate exercise test in a semi-supine position (60° head-up; ergoline GmbH, Germany). Such a semi-supine position was chosen to optimize patients comfort and safety (fainting) and to allow stable and high quality measurements by avoiding unnecessary upper-body movement due to maximal exercise. Semi-supine position allowed to use an arm sling to fix the left arm with the continuous blood pressure measurement (see below) on the level of the heart. At all locations, patients cycled at 60% of their maximal work rate assessed by an incremental ramp exercise test at 490 m until exhaustion. Data from incremental ramp exercise test have been published previously^11^. Patients were instructed to cycle at 60 rounds per minute (RPM) and were encouraged to continue as long as possible until RPM fell below 40 RPM for more than 5 s.

Work rate and breath-by-breath pulmonary gas exchange were recorded by a metabolic unit (Ergostick; Geratherm Medical AG, Germany) according to standard techniques^12^. Minute ventilation (${\dot{\text{V}}}_{{\text{E}}}$) and tidal volume (${\text{V}}_{{\text{T}}}$) were expressed in body temperature and pressure, saturated condition, and oxygen uptake (${\dot{\text{V}}}_{{{\text{O}}_{{2}} }}$) and carbon dioxide output (${\dot{\text{V}}}_{{{\text{CO}}_{{2}} }}$) in standard temperature and pressure, dry condition, according to convention. ${\dot{\text{V}}}_{{{\text{O}}_{{2}} }}$ predicted was calculated from Wasserman–Hansen prediction equations^13^. The increase in ${\dot{\text{V}}}_{{\text{E}}}$ due to the lower barometric pressure at 2048 m was adjusted as previously described^4^. Breathing reserve was calculated as 40 × FEV_1_ – ${\dot{\text{V}}}_{{\text{E}}}$ at end-exercise. Breath-by-breath changes in end-expiratory lung volume were monitored unobtrusively by calibrated respiratory inductive plethysmography operated in the direct-current mode (Respitrace; NIMS, Miami Beach, USA)^14,15^. Near-infrared spectroscopy optodes were placed bilaterally at the Fp1 and Fp2 landmarks of the 10-10 electrode placement system^16^ and bilaterally over the vastus lateralis muscles. Mean value of bilateral cerebral (CTO) and quadriceps muscle (MTO) tissue oxygenation, respectively, was calculated. Blood pressure was continuously monitored by the finger-cuff technique (Finometer Midi, FMS, The Netherlands) and calibration was validated by brachialis sphygmomanometric measurements. Finger pulse oximetry assessed arterial oxygen saturation (SpO_2_).

Arterial blood samples were drawn during rest and at end-exercise of each examination. Calculation of alveolar-arterial PO_2_ difference was performed as previously described^17^. Spirometries were performed according to standard techniques and reference values of the Global Lung Function Initiative (GLI) were applied^18–21^. Dyspnea and leg fatigue were rated on the Borg CR10 Scale^22^.

### Outcome

The main outcome was the difference in constant work-rate endurance time between tests performed after nights with NOT compared to nights after placebo at 2048 m. Secondary outcomes were changes in physiological response with exercise at 2048 m compared to 490 m, and NOT compared to placebo.

### Sample size and data analysis

To detect changes in endurance time between NOT and placebo with an effect size of 0.5, a power of 80% and an alpha level of 0.05, a sample size of a total of 32 patients was required. The data are summarized as means and standard errors. Analysis of the main outcome was performed in the intention-to-treat population with missing values replaced by multiple imputations using predictive mean matching with baseline variables as independent predictors^23^. Data from patients with complete data sets were included in the per-protocol approach analyzing the main and secondary outcomes. As a safety precaution, patients were not allowed to participate in exercise testing if they experienced predefined signs or symptoms of ARAHE or a resting SpO_2_ < 80% prior to the exercise testing. Baseline measurements at rest correspond to mean values during the last 3 min of a 10 min resting period on the bicycle preceding the actual constant work-rate exercise. End-exercise values were defined as mean values of the last 30 s before exhaustion i.e. before end of exercise.

Effects of altitude exposure and of treatment were assessed by computing linear mixed effects regression models with fixed effects of intervention (490 m, 2048 m—placebo, 2048 m—NOT) and random effects of individual patients. To determine low altitude predictors of constant work-rate endurance time at 2048 m, multivariable linear regression analysis with intervention (NOT, placebo), altitude (2048 m, 490 m), sex, intervention × altitude × sex, allocation sequence (A-D), age, BMI and FEV_1_% predicted was performed. Furthermore, due to the higher incidence of ARAHEs and non-random losses between placebo and NOT at 2048 m, exploratory post-hoc analysis was performed, simulating an exercise duration of 0 s when an ARAHE occurred. Statistical significance was assumed when P < 0.05 and 95% CIs of mean differences did not overlap zero.

## Results

Of 32 (17 women) randomized patients included in the intention-to-treat analysis, 22 (12 women) completed all exercise tests and could be included in the per-protocol analysis (Fig. 1). Reasons for protocol violations were the occurrence of ARAHEs in 9 of 32 (28%) patients during the exposure to 2048 m (8 during placebo treatment compared to 1 during NOT at 2048 m, P < 0.001)^10^. One additional patient refused exercise testing at 490 m. Otherwise, patients tolerated the exercise session well and no test had to be aborted.

Patient characteristics are summarized in Table 1. Patients were, mean ± SD, 66 ± 6 years old and had a mean FEV_1_ of 54 ± 13% predicted. Their maximal work rate achieved at 490 m was 90 ± 33 W, corresponding to 71 ± 22% predicted. The calculated 60% constant work-rate was 52 ± 20 W.

**Table 1:** Patient characteristics.

	Intention-to-treat population (N = 32)	Per-protocol population (N = 22)
Sex, male/female	15/17	10/12
Age, years	66 ± 6	66 ± 5
Body mass index, kg/m^2^	26.0 ± 4.5	25.7 ± 3.8
Current smokers, n (%)	8 (25)	5 (23)
Smoking pack-years	44 ± 27	43 ± 30
GOLD, grade 2/grade 3	22/10	17/5
**Pulmonary function and oxygen uptake**		
FVC, L	3.04 ± 0.88	3.00 ± 0.82
% predicted	84 ± 17	83 ± 17
FEV_1_, L	1.62 ± 0.60	1.64 ± 0.53
FEV, % predicted	54 ± 13	55 ± 13
FEV_1_/FVC	0.53 ± 0.11	0.53 ± 0.08
Maximal work rate, W	90 ± 33	96 ± 34
% predicted	71 ± 22	75 ± 22
${\dot{\text{V}}}_{{{\text{O}}_{{{2}^{{{\text{peak}}}} }} }}$, L/min	1.23 ± 0.37	1.28 ± 0.34
% predicted^1^	77 ± 21	83 ± 19
mL/min/kg	17.01 ± 4.57	17.98 ± 4.33
**Comorbidities, n (%)**		
Hypertension	12 (38)	10 (46)
Coronary artery disease	5 (16)	5 (22)
Diabetes mellitus	4 (13)	3 (14)
Depression	3 (9)	1 (5)
**Medications, n (%)**		
Inhaled glucocorticosteroids	14 (44)	9 (41)
Inhaled β-adrenergics	24 (75)	15 (68)
Inhaled anticholinergics	22 (69)	16 (73)
Diuretics	8 (25)	5 (23)
Antihypertensive medication	16 (50)	10 (45)
Antidiabetics	4 (13)	3 (14)
Antidepressants	6 (19)	5 (23)

Values are numbers (proportion) or mean ± SD. ^1^Normal values from Wasserman–Hansen were used for predicting V_O__2_^13^. *FEV*_*1*_ forced expiratory volume in the first second of expiration, *GOLD* Global Initiative for Chronic Obstructive Lung Disease, *FVC* forced vital capacity, ${\dot{\text{V}}}_{{{\text{O}}_{{{2}^{{{\text{peak}}}} }} }}$ maximal oxygen consumption.

### Main outcome

**Figure 2:**
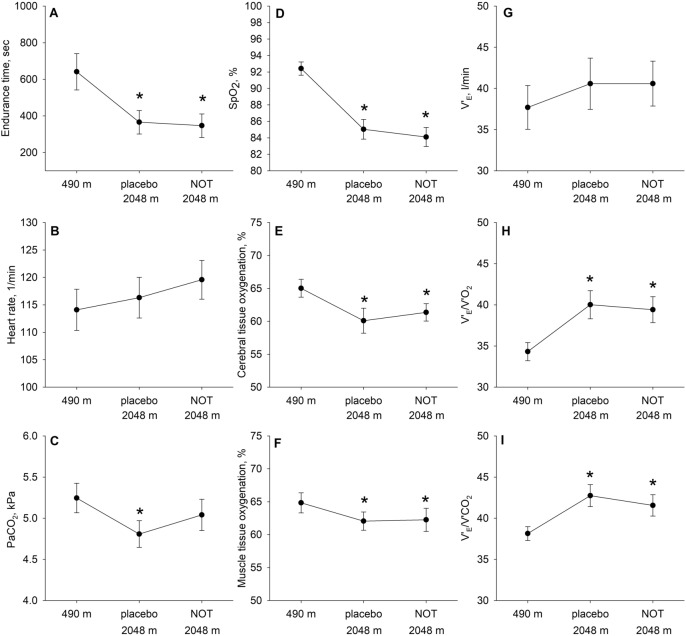
Exercise performance and physiological outcomes. Mean end-exercise values of main and secondary outcomes after different interventions (490 m, 2048 m with placebo, 2048 m with NOT). Panel (**A**) Endurance time; Panel (**B**) Heart rate; Panel (**C**) PaCO_2_; Panel (**D**) Pulse oximetry (SpO_2_); Panel (**E**) Cerebral tissue oxygenation; Panel (**F**) Muscle tissue oxygenation; Panel (**G**) Minute ventilation (${\dot{\text{V}}}_{{\text{E}}}$); Panel (**H**) ventilatory equivalent for oxygen uptake (${\dot{\text{V}}}_{{\text{E}}}$/${\dot{\text{V}}}_{{{\text{O}}_{{2}} }}$); Panel (**I**) Ventilatory equivalent for carbon dioxide output (${\dot{\text{V}}}_{{\text{E}}}$/${\dot{\text{V}}}_{{{\text{CO}}_{{2}} }}$); *p < 0.05 vs. corresponding value at 490 m, respectively.

In the intention-to-treat analysis, exercise duration was significantly reduced from 602 ± 65s at 490 m to 345 ± 62 s at 2048 m after placebo (Table 2, Fig. 2). The mean difference was − 258 s (95% CI, − 390 to − 125s, P < 0.001) and corresponded to a reduction in endurance time of − 35% (95% CI, − 47 to − 23%, P < 0.001) compared to 490 m. Exercise duration at 2048 m after NOT was 293 ± 60 s, therefore, exercise endurance after NOT and placebo was similarly reduced compared to 490 m, mean difference between NOT versus placebo, − 52 s (95% CI, − 174 to 70 s, P = 0.401). Findings were confirmed in the per-protocol analysis (Table 2) and in the worst-case scenario replacing missing exercise tests due to ARAHE by the value of 0 (mean treatment effect [95% CI] of − 1 s [− 119 to 117], P = 0.989).

**Table 2:** Performance and physiologic response to exercise at lowland and high altitude.

	490 m End exercise	2048 m—Placebo End exercise	2048 m—Placebo vs. 490 m, mean difference (95% CI)	2048 m—NOT End exercise	2048 m—NOT vs. 490 m, mean difference (95% CI)	Treatment effect, mean difference (95% CI)	P-value
PP: Endurance time, s	641 ± 76	366 ± 76^¶^	− 276 (− 395 to − 156)	347 ± 76	− 295 (− 414 to − 175)	− 19 (− 138 to 101)	0.757
PP: Endurance time, % 490 m	100	64 ± 5^¶^	− 36 (− 47 to − 26)	56 ± 5^¶^	− 44 (− 54 to − 33)	− 7 (− 18 to 3)	0.167
ITT: Endurance time, s	602 ± 65	345 ± 62^¶^	− 258 (− 390 to − 125)	293 ± 60	− 310 (− 435 to 185)	− 52 (− 174 to 70)	0.401
ITT: Endurance time, % 490 m	100	66 ± 5^¶^	− 35 (− 47 to − 23)	55 ± 4^¶^	− 46 (− 56 to − 35)	− 11 (− 23 to 1)	0.164
${\dot{\text{V}}}_{{{\text{O}}_{{2}} }}$, L/min	0.99 ± 0.03	0.92 ± 0.03	− 0.07 (− 0.15 to 0.01)	0.95 ± 0.03	− 0.04 (− 1.12 to 0.03)	0.03 (− 0.05 to 0.14)	0.498
${\dot{\text{V}}}_{{{\text{O}}_{{2}} }}$, % predicted^1^	59 ± 2	55 ± 2	− 4 (− 9 to 0)	56 ± 2	− 3 (− 8 to 1)	1 (− 4 to 6)	0.664
${\dot{\text{V}}}_{{{\text{CO}}_{{2}} }}$, L/min	0.90 ± 0.04	0.87 ± 0.04	− 0.03 (− 0.12 to 0.06)	0.90 ± 0.04	0.00 (− 0.09 to 0.09)	0.03 (− 0.06 to 0.12)	0.458
Respiratory Exchange Ratio	0.90 ± 0.01	0.93 ± 0.01^¶^	0.03 (0.00 to 0.07)	0.95 ± 0.01^¶^	0.05 (0.01 to 0.08)	0.01 (− 0.02 to 0.05)	0.455
Minute ventilation, L/min	37.7 ± 2.0	40.6 ± 2.0	2.9 (− 1.5 to 7.2)	40.6 ± 2.0	2.9 (− 1.5 to 7.2)	0.0 (− 4.3 to 4.4)	0.996
Tidal volume, L/min	1.3 ± 0.0	1.3 ± 0.0	0.0 (− 0.1 to 0.1)	1.3 ± 0.0	0.0 (− 0.1 to 0.1)	0.0 (− 0.1 to 0.1)	0.999
Breathing frequency, 1/min	29 ± 1	32 ± 1	3 (0 to 5)	32 ± 1	3 (0 to 5)	0 (− 2 to 2)	0.984
Breathing reserve, L/min	19.0 ± 3.5	17.6 ± 3.5	− 1.4 (− 6.6 to 3.8)	19.5 ± 3.5	0.6 (− 4.6 to 5.8)	1.9 (− 3.2 to 7.1)	0.455
Breathing reserve, %MVV	30 ± 3	29 ± 3	− 2 (− 9 to 6)	30 ± 3	− 1 (− 7 to 7)	2 (− 6 to 9)	0.680
Change in EELV, L	− 0.01 ± 0.17	0.29 ± 0.17	0.30 (− 0.08 to 0.68)	0.08 ± 0.17	0.09 (− 0.29 to 0.46)	− 0.21 (− 0.58 to 0.15)	0.648
${\dot{\text{V}}}_{{\text{E}}} \user2{ }$/${\dot{\text{V}}}_{{{\text{O}}_{{2}} }}$	34.3 ± 1.2	40.0 ± 1.2^¶^	5.7 (3.3 to 8.1)	39.4 ± 1.2^¶^	5.1 (2.7 to 7.5)	− 0.6 (− 3.0 to 1.8)	0.625
${\dot{\text{V}}}_{{\text{E}}} \user2{ }$/${\dot{\text{V}}}_{{{\text{O}}_{{2}} }}$ adj. to ${\text{P}}_{{\text{B}}}$ at 490 m	NA	35.8 ± 1.1	− 1.3 (− 1.0 to 3.5)	34.6 ± 1.1	0.2 (− 2.0 to 2.5)	− 1.0 (− 3.3 to 1.2)	0.374
${\dot{\text{V}}}_{{\text{E}}} \user2{ }$/${\dot{\text{V}}}_{{{\text{CO}}_{{2}} }}$	38.1 ± 1.1	42.7 ± 1.1^¶^	4.6 (2.7 to 6.5)	41.6 ± 1.1^¶^	3.4 (1.5 to 5.3)	− 1.2 (− 3.1 to 0.7)	0.224
${\dot{\text{V}}}_{{\text{E}}} \user2{ }$/${\dot{\text{V}}}_{{{\text{CO}}_{{2}} }}$, adj. to ${\text{P}}_{{\text{B}}}$ at 490 m	NA	38.0 ± 1.0	− 0.1 (− 2.0 to 1.8)	36.4 ± 1.0	− 1.7 (− 3.6 to 0.2)	− 1.6 (− 3.5 to 0.3)	0.100
SpO_2_, %	92 ± 1	85 ± 1^¶^	− 7 (− 9 to − 6)	84 ± 1^¶^	− 8 (− 10 to − 7)	− 1 (− 3 to 1)	0.251
Arterial pH	7.38 ± 0.00	7.41 ± 0.00^¶^	0.03 (0.01 to 0.05)	7.41 ± 0.00^¶^	0.03 (0.01 to 0.05)	− 0.00 (− 0.02 to 0.02)	0.777
PaCO_2_, kPa	5.3 ± 0.1	4.8 ± 0.1^¶^	− 0.5 (− 0.8 to − 0.2)	5.0 ± 0.1	− 0.3 (− 0.6 to 0.0)	0.2 (− 0.1 to 0.5)	0.195
PaO_2_, kPa	9.0 ± 0.2	7.0 ± 0.2^¶^	− 2.0 (− 2.4 to − 1.5)	6.8 ± 0.3^¶^	− 2.1 (− 2.7 to − 1.7)	− 0.2 (− 0.7 to 0.3)	0.398
SaO_2_, %	92 ± 1	84 ± 1^¶^	− 7 (− 10 to − 5)	83 ± 1^¶^	− 9 (− 11 to − 7)	− 2 (− 4 to 0)	0.106
DAaPO_2_, kPa	3.5 ± 0.2	2.5 ± 0.2^¶^	− 1.0 (− 1.4 to − 0.5)	2.5 ± 0.2^¶^	− 1.0 (− 1.5 to − 0.5)	0.0 (− 0.5 to 0.5)	0.995
Heart rate, bpm	114 ± 3	116 ± 3	2 (− 2 to 7)	119 ± 3	5 (0 to 10)	3 (− 2 to 8)	0.289
Heart rate reserve, bpm	40 ± 3	38 ± 3	− 2 (− 7 to 3)	35 ± 3	− 5 (− 10 to 0)	− 3 (− 8 to 2)	0.293
MAP, mmHg	127 ± 4	136 ± 4^¶^	9 (1 to 18)	139 ± 4^¶^	12 (3 to 22)	3 (− 6 to 12)	0.488
CTO, %	65 ± 1	61 ± 1^¶^	− 5 (− 8 to − 3)	61 ± 1^¶^	− 4 (− 6 to − 2)	1 (− 1 to 4)	0.265
MTO, %	65 ± 1	62 ± 1^¶^	− 3 (− 5 to − 1)	62 ± 1^¶^	− 3 (− 5 to − 1)	0 (− 2 to 2)	0.862
Borg CR10 Dyspnea	4.4 ± 0.4	5.1 ± 0.4	0.7 (− 0.2 to 1.6)	4.8 ± 0.4	0.5 (− 0.4 to 1.4)	− 0.3 (− 1.1 to 0.6)	0.577
Borg CR10 Leg fatigue	3.7 ± 0.3	4.1 ± 0.3	0.4 (− 0.4 to 1.3)	3.2 ± 0.4	− 0.5 (− 1.3 to 0.4)	− 0.9 (− 1.7 to − 0.5)	0.037

Total n = 22. Values are presented as mean ± SE; ^1^Normal values from Wasserman–Hansen were used for predicting ${{\text{V}}}_{{{\text{O}}_{{2}} }}$^13^; ^¶^P < 0.05 vs. the corresponding end-exercise value at 490 m. ${\dot{\text{V}}}_{{{\text{O}}_{{2}} }}$, oxygen uptake, ${\dot{\text{V}}}_{{{\text{CO}}_{{2}} }}$, carbon dioxide output; ${\dot{\text{V}}}_{{\text{E}}}$, minute ventilation; Breathing reserve calculated by (MVV – ${\dot{\text{V}}}_{{\text{E}}}$)/MVV × 100; EELV, EELV, end-expiratory reserve volume; ${\dot{\text{V}}}_{{\text{E}}}$/${\dot{\text{V}}}_{{{\text{O}}_{{2}} }}$, ${\dot{\text{V}}}_{{\text{E}}}$/${\dot{\text{V}}}_{{{\text{CO}}_{{2}} }}$, ventilatory equivalents for O_2_ uptake and CO_2_ output; ${\dot{\text{V}}}_{{\text{E}}}$/${\dot{\text{V}}}_{{{\text{CO}}_{{2}} }} \user2{ }$ adj. and ${\dot{\text{V}}}_{{\text{E}}}$/${\dot{\text{V}}}_{{{\text{O}}_{{2}} }}$ adj. to ${\text{P}}_{{\text{B}}}$ at 490 m, adjusted values that account for changes in barometric pressure of values expressed in BTPS (see “Methods” for explanation); SpO_2_, SaO_2_, arterial oxygen saturation by pulse oximetry and co-oximetry, respectively; DAaPO_2_, alveolar-arterial PO_2_ difference; MAP, mean blood pressure; CTO, cerebral tissue oxygenation; MTO, muscle tissue oxygenation.

### Secondary outcomes

A complete presentation of end-exercise values and mean differences between altitudes and interventions are provided in Table 2. Resting values are presented in the e-Table 1.

During the resting period at 2048 m after placebo, patients had lower values of arterial partial pressure of oxygen (PaO_2_), arterial oxygen saturation (SaO_2_) and arterial partial pressure of carbon dioxide (PaCO_2_) and increased resting pH and heart rate compared to 490 m (e-Table 1). CTO and MTO remained preserved. Patients rated their dyspnea sensation similar compared to rest at 490 m. NOT did not alter altitude-induced changes in these variables during the resting period.

Patients cycled for a shorter duration at 2048 m after placebo compared to 490 m and at end-exercise they were more hypoxemic and respiratory exchange ratios were higher than at 490 m, however, PaCO_2_ did not further decrease with exercise. Moreover, cerebral and muscular hypoxemia and worse ventilatory equivalents were observed compared to 490 m (Fig. 2, Table 2). However, breathing and heart rate reserves as well as dyspnea sensation and leg fatigue were similar (Table 2). After NOT, patients had similar altitude-induced changes in these variables compared to 490 m (Table 2, Fig. 2).

Multivariable regression analysis revealed that altitude but not the intervention (NOT, placebo) was an independent predictor of endurance time when controlled for sex, age, baseline FEV_1_, body-mass-index and allocation sequence (A–D) (Table 3).

**Table 3:** Multivariable regression model to assess predictors at 490 m of the exercise endurance time at 2048 m. Intention-to-treat population (N = 32).

Dependent: exercise duration, s	Coef	Std.Err	P value	95% CI
Men at 490 m (reference)	1437	713	0.044	38 to 2835
Δ as women at 490 m	− 31	124	0.803	− 275 to 213
Δ as men at 2048 m after placebo	− 228	91	0.013	− 408 to − 49
Δ as women vs men at 2048 m after placebo	− 55	130	0.670	− 311 to 200
Δ as men at 2048 m after NOT	− 309	87	< 0.001	− 480 to − 139
Δ as women vs men at 2048 m after NOT	− 1	128	0.993	− 252 to 250
Age, years	− 14	9	0.108	− 32 to 3
FEV_1_, %predicted	5	4	0.160	− 2 to 13
Body mass index, kg/m^2^	− 5	11	0.668	− 26 to 17
Allocation sequence (A–D)	− 13	46	0.773	− 103 to 77

*FEV*_*1*_ forced expiratory volume in the first second of expiration.

## Discussion

The main findings of the current study were that patients with moderate to severe COPD experience exercise impairments already at moderate altitude of 2048 m compared to 490 m and that NOT in the first night at altitude had no beneficial effect on next-day exercise performance compared to placebo. The reduced exercise endurance was associated with pronounced arterial hypoxemia, cerebral and muscular hypoxia, ventilatory limitations, a higher respiratory exchange ratio, higher ventilatory equivalents for oxygen uptake and carbon dioxide output and a higher systemic blood pressure compared to 490 m.

Data on oxygen therapy at altitude in COPD patients and the consequences on their exercise performance are scant. Highlanders with COPD residing at Bogota (2640 m) showed an improvement of 35% in a constant work-rate exercise test whilst using oxygen supplementation which augmented the arterial oxygen content and ventilatory efficiency at end-exercise^24^. Furthermore, dyspnea was alleviated when comparing isotime values between exercise with oxygen and ambient air. However, during mountain activities, the administration of supplemental oxygen during exercise is cumbersome and impractical. NOT would be an alternative. In the current study, NOT improved nocturnal hypoxemia, sleep apnea, subjective sleep quality and reduced the incidence of ARAHEs compared to placebo^10^. A randomized clinical trial in 23 patients with pulmonary hypertension, a comorbidity promoted by COPD, showed that one week of NOT can improve the six-minute walk distance compared to placebo (mean difference [95% CI] + 25 m [3–46 m])^25^. Since we assumed that the deleterious effects of altitude were pronounced after one night in hypoxic conditions due to symptoms of acute mountain sickness, nocturnal sleep-disordered breathing and worse subjective sleep quality, we expected that NOT would improve daytime exercise performance compared to placebo. However, NOT had no protective effect on the various exercise-limiting factors resulting in exercise intolerance compared to placebo despite the beneficial effects on sleep disordered breathing and ARAHE^10^.

The altitude-induced reduction in exercise performance of 35% (258 s) after placebo, and 46% (310 s) after NOT at 2048 m exceeded the clinically minimal important difference of 46–105 s proposed in COPD patients^26^. Our results are in accordance with previously reported reductions of 54% in 31 patients with COPD exposed to 2590 m^4^, indicating pronounced vulnerability to hypobaric hypoxia. To our knowledge, no other randomized trial investigating exercise performance and exercise-limiting factors in COPD travelling to high altitude have been published. Another study evaluating time to exhaustion in 8 elite cyclists reported a linear reduction of 14.3%/1000 m of altitude gain between 800 to 2800 m^27^. These comparisons suggest that exercise performance in patients with COPD may be more compromised at moderate altitude compared to athletes, however, differences in training status, age, altitude and study design make comparisons between these studies in healthy, athletic individuals and COPD difficult.

Exercise-limiting factors in COPD near sea level are multifactorial and have been reviewed previously^2^. Less known are the exercise-limiting factors at altitude in COPD. A previous study conducted at 2590 m in patients with COPD revealed pronounced hypoxemia despite increased ${\dot{\text{V}}}_{{\text{E}}}$ at end-exercise compared to 490 m^4^. The higher ${\dot{\text{V}}}_{{\text{E}}}$ was associated with lower PaCO_2_, indicating that hypoxemia induced hyperventilation. However, the hyperventilation caused ventilatory inefficiency for oxygen uptake and CO_2_ output and pronounced dyspnea sensation at 2590 m compared to 490 m. Furthermore, due to pronounced hypoxemia, exercise-induced cerebral tissue hypoxia at 2590 m was observed compared to sustained cerebral oxygenation at 490 m. We confirmed these findings in cerebral deoxygenation in our patients at 2048 m (61% versus 65% at 490 m P < 0.05, compared to 55% reported at 2590 m), suggesting that cerebral hypoxia might have contributed to the observed exercise intolerance at 2048 m compared to 490 m.

The decrease of MTO during exercise was more pronounced at 2048 m compared to 490 m, which might have contributed to the reduced exercise endurance. In the previous study at 2590 m, the MTO decreased similarly as at 490 m, although, the decrease tended to be more distinctive at 2590 m (66% to 53% rest to end-exercise) compared to 490 m (68% to 59%), P < 0.05 altitude vs. 490 m for both comparisons. A possible explanation could be the shorter exercise time at 2590 m (202 s compared to 366 s at 2048 m), which might have prevented excessive muscle tissue deoxygenation with exercise.

The current results should not be extrapolated to patients with more severe COPD or higher altitudes. A duration of one night of NOT might not have been enough to improve exercise performance, however, effects of NOT during a prolonged high altitude stay remain unknown. The higher incidence of ARAHEs during sojourns with placebo versus NOT (8 vs 1, P < 0.001) and thus, the non-random loss of patients, might have led to a ‘survivor’ bias potentially impacting the robustness of our conclusions. However, findings remained robust and were independent of the analysis strategy (by intention-to-treat, per-protocol or post-hoc worst-case scenario analysis). The three analysis strategies revealed upper 95% confidence intervals of the treatment effect with NOT of 70, 101 and 117 s, respectively. This indicates that it is likely that NOT does not improve endurance time by a clinically meaningful amount of 105 s^26^.

## Interpretation

In the current randomized clinical trial, we observed an impaired exercise endurance in patients with moderate to severe COPD at moderate altitude mediated by arterial and cerebral hypoxia, impaired gas exchange and ventilatory constraints. NOT at 2048 m failed to improve next-day exercise performance compared to placebo. However, NOT improved nocturnal sleep-disordered breathing, subjective sleep quality and reduced the incidence of ARAHEs compared to placebo, allowing more patients with COPD to remain and exercise at high altitude. Therefore, COPD patients may benefit from NOT during a short sojourn at high altitude. Whether the effects of NOT during a prolonged stay would improve daytime exercise performance and other outcomes remains to be elucidated.

## Supplementary Information

Supplementary Table 1.

## Data Availability

Anonymized data underlying this study can be requested by qualified researchers.
