# Supplementary material for: Effect of nocturnal oxygen therapy on exercise performance of COPD patients at 2048 m: data from a randomized clinical trial
Source: Sci Rep. 2021 Oct 13;11:20355. doi: 10.1038/s41598-021-98395-w (PMC8514448; doi:10.1038/s41598-021-98395-w)
Supplement: Supplementary file 1 — Supplementary Table 1. [file 41598_2021_98395_MOESM1_ESM.docx]

**Supplemental material**

**-**

**Effect of Nocturnal Oxygen Therapy on Exercise Performance of COPD patients at 2048 m - Data from a Randomized Clinical Trial**

Sophia Gutweniger^1^, Tsogyal D. Latshang^1^, MD, Sayaka S. Aeschbacher^1^, MD, Fabienne Huber^1^, MD, Deborah Flueck^1^, MD, Mona Lichtblau^1^, MD, Stefanie Ulrich^1^, MD, Elisabeth D. Hasler^1^, MD, Philipp M. Scheiwiller^1^, MD, Silvia Ulrich^1^, Konrad E. Bloch^1^, MD, Michael Furian^1^, PhD

^1^Department of Respiratory Medicine, University Hospital of Zurich, Zurich, Switzerland

**Correspondence**

Michael Furian, Dr. sc. ETH

University Hospital Zurich, Department of Respiratory Medicine,

Raemistrasse 100, CH 8091 Zurich, Switzerland

Phone: +41 44 255 38 28

Fax: +41 255 44 51

e-mail: michael.furian@usz.ch

**e-Table 1.** Resting values at lowland and high altitude.

|  | 490m rest | 2048m – Placebo rest | 2048m – Placebo vs. 490m, mean difference at rest (95% CI) | 2048m – NOT rest | 2048m – NOT vs. 490m, mean difference at rest (95% CI) | Treatment effect,  mean difference (95% CI) | P-value |
| --- | --- | --- | --- | --- | --- | --- | --- |
| V’O_2_, l/min | 0.27±0.03 | 0.29±0.04 | 0.03 (-0.05 to 0.10) | 0.29±0.03 | 0.03 (-0.05 to 0.10) | 0.00 (-0.1 to 0.1) | 0.979 |
| V’O_2_, % predicted | 16±2 | 17±2 | 2 (-3 to 6) | 17±2 | 2 (-3 to 6) | -0.06 (-4.6 to 4.5) | 0.978 |
| V’CO_2_, l/min | 0.21±0.04 | 0.23±0.04 | 0.02 (-0.06 to 0.11) | 0.23±0.04 | 0.03 (-0.06 to 0.11) | 0.02 (-0.09 to 0.09) | 0.957 |
| Respiratory exchange ratio | 0.79±0.01 | 0.79±0.01 | 0.01 (-0.03 to 0.04) | 0.80±0.01 | 0.01 (-0.02 to 0.05) | 0.01 (-0.03 to 0.04) | 0.743 |
| Minute ventilation, l/min | 10.9±2.0 | 12.3±2.0 | 1.3 (-3.0 to 5.7) | 12.2±2.0 | 1.3 (-3.0 to 5.6) | -0.1 (-4.4 to 4.3) | 0.979 |
| Tidal volume, l/min | 0.6±0.0 | 0.7±0.0 | 0.0 (0.0 to 0.1) | 0.7±0.0 | 0.0 (-0.1 to 0.1) | 0.0 (-0.1 to 0.1) | 0.872 |
| Breathing frequency, 1/min | 18±1 | 19±1 | 1 (-1 to 3) | 19±1 | 1 (-1 to 4) | 0 (-2 to 3) | 0.851 |
| Breathing reserve, l/min | 45.7±3.5 | 45.9±3.5 | 0.2 (-5.0 to 5.4) | 47.9±3.5 | 2.2 (-3.0 to 7.4) | 2.0 (-3.1 to 7.1) | 0.439 |
| Breathing reserve, %MVV | 79±3 | 77±3 | -3 (-10 to 5) | 78±4 | -1 (-8 to 6) | 1 (-6 to 8) | 0.731 |
| V’_E_/V’O_2_ | 34.6±1.2 | 35.7±1.2 | 1.1 (-1.3 to 3.6) | 34.8±1.2 | 0.2 (-2.2 to 2.6) | -0.9 (-3.4 to 1.5) | 0.460 |
| V’_E_/V’O_2_ adj. to PB at 490m | NA | 34.38±1.1 | -0.2 (-2.5 to 2.1) | 33.9±1.1 | -0.7 (-3.0 to 1.5) | -0.5 (-2.8 to 1.8) | 0.661 |
| V’_E_/V’CO_2_ | 43.9±1.1 | 44.9±1.1 | 1.0 (-0.9 to 2.9) | 43.6±1.1 | -0.4 (-2.3 to 1.5) | -1.4 (-3.3 to 0.5) | 0.160 |
| V’_E_/V’CO_2_, adj. to PB at 490m | NA | 43.3±1.0 | -0.7 (-2.6 to 1.2) | 42.4±1.0 | -1.5 (-3.4 0.4) | -0.8 (-2.7 to 1.1) | 0.386 |
| SpO_2_, % | 95±1 | 91±1¶ | -4 (-5 to -2) | 91±1¶ | -4 (-5 to -2) | 0 (-2 to 2) | 0.964 |
| Arterial pH | 7.44±0.0 | 7.47±0.0¶ | 0.03 (0.01 to 0.05) | 7.47±0.00¶ | 0.03 (0.01 to 0.05) | -0.00 (-0.02 to 0.02) | 0.993 |
| PaCO_2_, kPa | 4.9±0.1 | 4.6±0.1¶ | -0.4 (-0.7 to -0.1) | 4.6±0.1¶ | -0.3 (-0.6 to -0.8) | 0.1 (-0.2 to 0.3) | 0.714 |
| PaO_2_, kPa | 9.0±0.2 | 7.9±0.2¶ | -1.1 (-1.6 to -0.7) | 7.8±0.2 | -1.3 (-1.7 to -0.8) | -0.1 (-0.5 to 0.3) | 0.627 |
| SaO_2_, % | 94±1 | 90±1¶ | -4 (-6 to -2) | 89±1¶ | -4 (-6 to -2) | -1 (--2 to 1) | 0.638 |
| DAaPO_2_, kPa | 3.9±0.2 | 1.9±0.2¶ | -1.9 (-2.3 to -1.5) | 2.0±0.2¶ | -1.9 (-2.3 to -1.5) | 0.0 (-0.4 to 0.4) | 0.870 |
| Base excess | 0.7±0.6 | 1.0±0.6 | 0.2 (-1.0 to 1.5) | 1.2±0.6 | 0.5 (-0.8 to 1.7) | 0.2 (-1.0 to 1.5) | 0.688 |
| Heart rate, bpm | 73±3 | 80±3¶ | 7 (2 to 12) | 79±3¶ | 7 (2 to 12) | -1 (-6 to 5) | 0.846 |
| Heart rate reserve, bpm | 81±3 | 74±3¶ | -7 (-12 to -2) | 74±3¶ | -7 (-12 to -2) | 1 (-5 to 6) | 0.846 |
| O_2_ pulse, ml/beat | 3.7±0.4 | 3.7±0.4 | 0.0 (-0.7 to 0.7) | 3.8±0.4 | 0.0 (-0.6 to 0.7) | 0.0 (-0.6 to 0.7) | 0.888 |
| MAP, mmHg | 98±4 | 101±4 | 4 (-5 to 13) | 100±4 | 2 (-7 to 12) | -1 (-11 to 8) | 0.761 |
| CTO, % | 65±1 | 64±1 | -1 (-4 to 1) | 64±1 | -1 (-3 to 1) | 0 (-2 to 3) | 0.737 |
| MTO, % | 71±1 | 71±1 | 1 (-2 to 3) | 71±1 | 1 (-2 to 3) | 0 (-2 to 2) | 0.957 |
| Borg CR10 dyspnea | 0.6±0.4 | 0.5±0.4 | -0.3 (-0.9 to 0.8) | 0.4±0.4 | -0.2 (-1 to 0.7) | -0.1 (-1.0 to 0.7) | 0.756 |
| Borg CR10 leg fatigue | 0.3±0.3 | 0.3±0.4 | 0.0 (-0.8 to 0.9) | 0.0±0.3 | -0.2 (-1.1 to 0.6) | -0.3 (-1.1 to 0.5) | 0.483 |
| Total n=22. Values are presented as mean±SE; ¶ p<0.05 vs. the corresponding rest value at 490 m. V’O2, oxygen uptake, V’CO2, carbon dioxide output; RER, respiratory exchange ratio; V’E, minute ventilation; VT, tidal volume; Bf, breathing rate; BR, breathing reserve calculated by (MVV-V’E)/MVV*100; EELV, end-expiratory reserve volume; IRV, inspiratory reserve capacity; V’E/V’O2, V’E/V’CO2, ventilatory equivalents for O2 uptake and CO2 output; V′E/V′CO2 adj. and V′E/V′O2 adj. to PB at 490 m, adjusted values that account for changes in barometric pressure of values expressed in BTPS (see Methods for explanation); SpO2, SaO2, arterial oxygen saturation by pulse oximetry and co-oximetry, respectively; DAaPO2, alveolar-arterial PO2 difference; MAP, mean blood pressure; CTO, cerebral tissue oxygenation; MTO, muscle tissue oxygenation. | | | | | | | |
